# An Observational Study of the Etiology, clinical presentation and outcomes associated with peritonitis in Lilongwe, Malawi

**DOI:** 10.1186/1749-7922-6-37

**Published:** 2011-11-08

**Authors:** Jonathan C Samuel, Javeria S Qureshi, Gift Mulima, Carol G Shores, Bruce A Cairns, Anthony G Charles

**Affiliations:** 1 Department of Surgery, Kamuzu Central Hospital, PO Box 149, Lilongwe, Malawi; 2 Department of Surgery, University of North Carolina, 4001 Burnett Womack Bldg, CB 7050, Chapel Hill, NC, 27599 USA; 3 Lineberger Comprehensive Cancer Center, University of North Carolina, CB 7295, Chapel Hill, NC, 27599 USA; 4 Department of Otolaryngology/Head & Neck Surgery, University of North Carolina, 170 Manning Drive, CB 7070, Physician's Office Bldg G-190, Chapel Hill, NC, 27599 USA; 5 NC Jaycee Burn Center, University of North Carolina, 101 Manning Drive, CB 7600, Chapel Hill, NC, 27599 USA

## Abstract

**Introduction:**

Peritonitis is a life-threatening condition with a multitude of etiologies that can vary with geographic location. The aims of this study were to elucidate the etiology, clinical presentation and outcomes associated with peritonitis in Lilongwe, Malawi.

**Methods:**

All patients admitted to Kamuzu Central Hospital (KCH) who underwent an operation for treatment of peritonitis during the calendar year 2008 were eligible. Peritonitis was defined as abdominal rigidity, rebound tenderness, and/or guarding in one or more abdominal quadrants. Subjects were identified from a review of the medical records for all patients admitted to the adult general surgical ward and the operative log book. Those who met the definition of peritonitis and underwent celiotomy were included.

**Results:**

190 subjects were identified. The most common etiologies were appendicitis (22%), intestinal volvulus (17%), perforated peptic ulcer (11%) and small bowel perforation (11%). The overall mortality rate associated with peritonitis was 15%, with the highest mortality rates observed in solid organ rupture (35%), perforated peptic ulcer (33%), primary/idiopathic peritonitis (27%), tubo-ovarian abscess (20%) and small bowel perforation (15%). Factors associated with death included abdominal rigidity, generalized (versus localized) peritonitis, hypotension, tachycardia and anemia (p < 0.05). Age, gender, symptoms (obstipation, vomiting) and symptom duration, tachypnea, abnormal temperature, leukocytosis, hemoconcentration, thrombocytopenia and thrombocytosis were not associated with mortality (p = NS).

**Conclusions:**

There are several signs and laboratory findings predictive of poor outcome in Malawian patients with peritonitis. Tachycardia, hypotension, anemia, abdominal rigidity and generalized peritonitis are the most predictive of death (P < 0.05 for each). Similar to studies from other African countries, in our population the most common cause of peritonitis was appendicitis, and the overall mortality rate among all patients with peritonitis was 15%. Identified geographical differences included intestinal volvulus, rare in the US but the 2^nd ^most common cause of peritonitis in Malawi and gallbladder disease, common in Ethiopia but not observed in Malawi. Future research should investigate whether correction of factors associated with mortality might improve outcomes.

## Introduction

Peritonitis is a common surgical emergency with a high mortality rate ranging from 10-60% depending on the study [1]. Though there are several distinct causes of peritonitis, prompt recognition and surgical treatment has been the mainstay of therapy since Kirschner first reported improved outcomes with surgical intervention [2]. This treatment approach requires both a knowledge of the signs and symptoms of peritonitis to aid diagnosis and an understanding of common causes to assist the surgeon in appropriate surgical care.

Despite a high prevalence of peritonitis reported in several African countries [3-5], little is known about the presentation, causes, and outcome of peritonitis in the south eastern African country of Malawi. Local environmental factors combined with genetic predispositions lead to marked variation in disease cause and presentation, and defining this can lead to improved local care and better overall understanding of the disease process. Like many resource-poor settings, acutely ill patients in Malawi often present late in the disease process and there is frequently limited time for diagnostic studies prior to definitive therapy. This knowledge gap, high morbidity and mortality, and delayed presentation illustrates a problem that has potential for improvement in care through a better ability to recognize and treat peritonitis. Therefore, the goals of this study were to better elucidate the etiology, presentation (history, physical, laboratory and ultrasound findings) and outcomes associated with peritonitis at a single large referral hospital in Lilongwe, Malawi.

## Methods

### Study Setting

This study was conducted at Kamuzu Central Hospital (KCH) in Lilongwe, the capital of Malawi, during the calendar year 2008. KCH is the 830-bed referral hospital for the central region of Malawi, serving a population of around 5 million people. The hospital has a 24-h casualty department, 4-bed intensive care unit, 4-bed high dependency unit, several open wards each with capacity for around 50 surgical patients, radiology department with plain radiography and limited ultrasound capabilities, and four operating rooms. The hospital lacked pathology capabilities in 2008 and hospital laboratory testing is limited to complete blood count, with more extensive testing available in only limited circumstances through off-site private laboratories. An on-site blood bank supplies whole blood and packed red blood cells, with occasional availability of plasma or platelets.

### Subject Identification and Data Acquisition

All patients admitted to KCH who underwent an operation for treatment of peritonitis during the calendar year 2008 were eligible. Peritonitis was defined as abdominal rigidity, rebound tenderness, and/or guarding in one or more abdominal quadrants. Subjects were identified retrospectively through a review of all medical records of patients cared for in 2008 on the adult general surgical wards (approximately 5000) and from the operative log book. Data was collected from the medical record and included: gender, age, date of admission, date of surgery, date of discharge or death, surgical procedure and operative diagnosis, date of onset and type of symptoms, presence of guarding, rebound, or rigidity and abdominal quadrant(s) affected, vital signs on presentation including temperature, heart rate, blood pressure, and respiratory rate, date and results of initial complete blood count if performed, and results of abdominal ultrasound if performed. Data was collected and entered into a Microsoft Excel spreadsheet (Office 2007) and analyzed using Stata (version 11).

### Analysis of Data

Descriptive statistics were calculated for the following: operative diagnosis; overall and diagnosis-specific mortality rates; age and gender distributions; time (in days) from symptom onset, presentation, and outcome (death versus discharge); presence of rigidity; localized versus generalized peritonitis; presenting vital signs including systolic blood pressure (< 90, ≥ 90), respiratory rate (< 30, ≥ 30), heart rate (< 100, ≥ 100), and temperature (< 35.5, 35.5-38.4, > 38.4); Complete blood count results including total leukocyte count (< 4, 4-11, > 11) hematocrit (< 31.6, 31.6-47.9, > 47.9), and platelet count (< 100, 100-399, ≥400); and ultrasound findings if performed (presence or absence of free fluid, abscess, and/or appendicitis).

Correlations between outcome (death during hospitalization versus discharge) and clinical data (age, gender, type of symptoms and symptom duration, examination findings, vital signs, and laboratory values) were calculated using chi-squared analyses. In comparison to operative diagnosis the sensitivity and specificity of ultrasound in diagnosing appendicitis and free fluid/abscess was reported.

## Results

We identified 190 subjects meeting the definition of peritonitis who underwent celiotomy. Sixty-nine percent were male. The average age was 35 (median 32, range 10-84). The youngest subject was 10, and 10 subjects were under the age of 18. The most common etiologies were appendicitis (22%), intestinal volvulus (17%), perforated peptic ulcer (11%) and small bowel perforation (11%) (table 1). The overall mortality rate associated with peritonitis was 15%, with the highest mortality rates observed in solid organ rupture (35%), perforated peptic ulcer (33%), primary/idiopathic peritonitis (27%), tubo-ovarian abscess (20%) and small bowel perforation (15%) (table 1). Factors associated with increased mortality include abdominal rigidity, generalized peritonitis (versus localized peritonitis), hypotension, tachycardia and anemia (p < 0.05); age, gender, symptoms (obstipation, vomiting) and symptom duration, tachypnea, abnormal temperature, hemoconcentration, thrombocytopenia and thrombocytosis were not associated with increased mortality (p = NS) (table 2).

**Table 1:** Etiology of peritonitis in relation to gender, age, and in-hospital mortality.

Diagnosis	Number	Male	Female	Mortality
Appendicitis	42 (22%)	30 (71%)	12 (29%)	2.4% (1/42)

Intestinal Volvulus*	32 (17%)	30 (94%)	2 (6.3%)	9.4% (3/32)

Perforated Peptic Ulcer†	21 (11%)	19 (90%)	2 (9.5%)	33% (7/21)

Small Bowel Perforation	20 (11%)	15 (75%)	3 (15%)	15% (3/20)

Solid Organ Rupture	17 (8.9%)	9 (53%)	8 (47%)	35% (6/17)

Primary/Idiopathic	15 (7.9%)	8 (53%)	7 (47%)	27% (4/15)

Ischemic Bowel‡	12 (6.3%)	5 (42%)	7 (58%)	8.3% (1/12)

Intussusception	8 (4.2%)	5 (63%)	3 (38%)	0% (0/8)

Tubo-Ovarian Abscess	5 (2.6%)	na	5 (100%)	20% (1/5)

Bowel Obstruction	5 (2.6%)	1 (20%)	4 (80%)	0% (0/5)

All Other§	13 (6.8%)	9 (69%)	4 (31%)	15% (2/13)

Total	190 (100%)	131 (69%)	57 (30%)	15% (28/190)

*Sigmoid volvulus (23), Mid-gut Volvulus (9)

†Duodenal (14), Gastric (7)

‡ischemic bowel not otherwise due to bowel obstruction or volvulus

§Colorectal (3), Postoperative (3), Small Bowel Cancer (2), hernia (2), TB (1), Pancreatitis (1), Traumatic Gastric Perforation (1)

**Table 2:** Association between presentation and outcome.

Presenting Factor		Death	Discharge	p value (χ^2^)
Age	< 50	21	133	

	≥50	7	27	0.303

Gender	Male	18	113	

	Female	10	47	0.501

Symptom Duration	< 4 days	12	79	

	≥4 days	10	75	0.776

Obstipation	Yes	8	63	

	No	16	93	0.511

Vomiting	Yes	7	69	

	No	17	87	0.164

Rigidity	Yes	10	36	

	No	13	122	0.033

Peritonitis	Localized	0	34	

	Generalized	23	124	0.014

Blood Pressure	≥90	24	152	

	< 90	3	2	0.004

Respiratory Rate	< 30	4	62	

	≥30	4	17	0.073

Heart Rate	< 100	3	60	

	≥100	24	93	0.005

Temperature	35.5-38.4	6	48	

	< 35.5 or > 38.4	2	10	0.593

Leukocytosis	4-11	6	60	

(WBC*10^4^/μL)	< 4 or > 11	12	44	0.056

Anemia	> 31.5	9	84	

(Hematocrit, %)	≤31.5	9	20	0.005

Hemoconcentration	< 48	14	84	

(Hematocrit, %)	≥48	4	20	0.768

Thrombocytopenia	≥100	14	96	

(Platelets*10^4^/μL)	< 100	4	8	0.056

Thrombocytosis	< 400	16	96	

(Platelets*10^4^/μL)	≥400	2	8	0.625

Preoperative ultrasound was performed in 51 of the 190 cases of peritonitis. Of the 51 ultrasounds, 22 were performed to evaluate for appendicitis and 23 were performed to evaluate for fluid and/or abscesses. A comparison between ultrasound results and intra-operative findings revealed a sensitivity and specificity for appendicitis was 0.5 and 1.0, and for fluid and/or abscess 0.82 and 0.83, respectively (table 3).

**Table 3:** Comparison between ultrasound results and intra-operative findings.

Ultrasound for Appendicitis		Intraoperative Finding		
		**Appendicitis**	**No Appendicitis**	

Ultrasound Finding	Appendicitis	9	0	

	No Appendicitis	9	4	

Ultrasound for Fluid/Abscess		Intraoperative Finding		

		Fluid/Abscess	No Fluid/Abscess	

Ultrasound Finding	Fluid/Abscess	14	1	

	No Fluid/Abscess	3	5	

## Discussion

This study outlines the etiology, associated presenting signs and symptoms, and outcomes of surgically managed peritonitis in a tertiary care center in central Malawi. The most common etiologies of peritonitis were appendicitis and volvulus. Abdominal rigidity, generalized peritonitis (versus localized), hypotension, tachycardia and anemia were significantly associated with mortality. The overall mortality rate was 15%. Ultrasound was specific but not sensitive in diagnosing appendicitis.

There are several similarities between our findings and those from other African countries. Appendicitis was the most common cause of peritonitis in our series (21%) and in studies on acute abdomen from Ghana (43.1%), Nigeria (40.3%) and Ethiopia (24.5%)[3,5,6]. One important distinction is that our study included patients with peritonitis defined as rigidity, guarding, or rebound tenderness, while these other studies included all patients with acute abdomen. Nega (2009) was the only investigator to report specific symptoms and reported guarding in only 39% of his patients, and though tenderness was present in 78% this was not specifically peritoneal tenderness.

Our mortality rate (15%) was similar to reported rates from Ethiopia (4.9-15.3%)[5,7]. A report from the 1960s in England also had a similar mortality rate of 20%, though this study included only patients with generalized peritonitis [8]. Interestingly, we found no correlation between the duration of symptoms and mortality, while Kotiso *et al*. noted 7.6% mortality rate in patients with symptoms of 2 days or less, compared with 25% among those with symptoms over 2 days in duration [7]. Though it is unclear why we did not observe a similar trend, one hypothesis is that our population had more "survivor bias" with the sickest dying prior to presentation; this bias is noted in a variety of epidemiologic studies from developing countries [9-11].

We found a significant correlation between outcome and several presenting signs and laboratory values. Generalized peritonitis (versus localized) was correlated with mortality. This is likely because localized peritonitis was most commonly seen in appendicitis which had a low mortality rate, whereas all cases of perforated peptic ulcer (with a high mortality rate) had generalized peritonitis (data not shown). We also found that hypotension, tachycardia, and anemia were associated with increased mortality. Several of these factors are also predictive of mortality in other surgical emergencies including traumatic injuries and necrotizing soft tissue infections [12,13]. Triage and care of patients with peritonitis might therefore be improved by using predictive tools similar to those applied to other acute surgical conditions such as trauma and necrotizing soft tissue infections.

Surgical diseases leading to peritonitis have a geographic variability. For example, in developed countries diverticulitis is a common cause of peritonitis, while we did not observe any cases of diverticulitis in our series [8]. Additionally, we noted variation even within Africa, as several studies from Ethiopia report gallbladder pathology including gangrenous cholecystitis and gallbladder empyema whereas in our study there was no gallbladder pathology [7,14].

The specificity of ultrasound (1.0) among those suspected of having appendicitis was similar to a that reported in a meta-analysis of ultrasound for appendicitis in adults (0.93), however our calculated sensitivity was considerably lower than the meta-analysis (0.50 versus 0.83) [15]. One potential explanation of why we observed a lower than expected sensitivity is the selection bias in our study related to including only patients with peritonitis, as it may be that appendicitis is more difficult to diagnose when peritonitis is present due to patient intolerance of the examination or less reliable ultrasound findings late in the disease process. An alternative explanation is that the test is operator-dependent and the ultrasonographers are not experienced or adequately trained to diagnose appendicitis. Regardless inability to diagnose appendicitis by ultrasound in patients with peritonitis did not sway the clinician away from surgical intervention. In this study, the sensitivity and specificity of ultrasound to detect free fluid and/or abscess was 0.82 and 0.83. Interpretation of this is limited without intra-operative quantification of fluid volume, as prior studies suggest a minimum of 100 mL to over 500 mL of fluid is necessary for an ultrasound to reveal fluid [16].

This retrospective study was not able to examine the utility of plain films in the management of peritonitis because patients often keep their radiographs upon discharge. Our analysis also showed association between preoperative factors and outcome, but this observational study does not prove causality. Future research should aim to determine if correction of factors associated with mortality (such as fluid resuscitation to correct tachycardia and/or hypotension) might improve outcomes. The generalizability of this study is also limited to adult patients at a tertiary care setting, as we did not include patients admitted to the pediatric ward or patients managed in district hospitals or health centers. Lastly, the definition of peritonitis, though standardized, assumes that all health care providers are adept at assessing the abdominal exam for guarding, rebound tenderness, and rigidity.

## Conclusions

In our setting peritonitis is associated with an overall mortality rate of 15%. The most common causes are appendicitis and volvulus, and factors associated with death include abdominal rigidity, generalized (versus localized) peritonitis, hypotension, tachycardia and anemia. Future research should investigate whether preoperative correction of these factors improves survival.

## List of abbreviations

KCH: Kamuzu Central Hospital;

## Competing interests

The authors declare that they have no competing interests.

## Authors' contributions

Author contributions were as follows: Conception and design (JS); acquisition of data (JS, GM); analysis and interpretation of data (JS); drafting of the manuscript (JS, JQ, GM); critical revision of the manuscript (CS, BC, AC). All authors read and approved the final manuscript.
